# Regulatory Mechanisms of Lipid Rafts in Remodeling the Tumor Immune Microenvironment of Colorectal Cancer and Targeted Therapeutic Strategies

**DOI:** 10.3390/biom15121675

**Published:** 2025-12-01

**Authors:** Zhihong Cheng, Jian Gu, Yaoyao Lu, Mingdong Cai, Tao Zhang, Jiliang Wang

**Affiliations:** 1 Department of Gastrointestinal Surgery, Union Hospital, Tongji Medical College, Huazhong University of Science and Technology, Wuhan 430022, China; 2 Department of Anesthesiology, Union Hospital, Tongji Medical College, Huazhong University of Science and Technology, Wuhan 430022, China; 3 Key Laboratory of Anesthesiology and Resuscitation, Huazhong University of Science and Technology, Ministry of Education, Wuhan 430022, China; 4 Institute of Anesthesia and Critical Care Medicine, Union Hospital, Tongji Medical College, Huazhong University of Science and Technology, Wuhan 430022, China

**Keywords:** lipid rafts, colorectal cancer, tumor immune microenvironment, targeted therapy, cholesterol metabolism

## Abstract

Immunotherapy has demonstrated significant efficacy in colorectal cancer (CRC), but its therapeutic effects remain limited in microsatellite stable (MSS) patients, indicating the critical role of the tumor immune microenvironment (TIME) in regulating immune responses. Lipid rafts, dynamic membrane microdomains enriched in cholesterol and sphingolipids, have emerged as potential targets for TIME remodeling through their integration of immune signal transduction, enrichment of cell death receptors, and regulation of immune cell functionality. This review outlines the pivotal mediating roles of lipid rafts in cellular survival, death, and tumor progression. Specifically, MSS-type CRC exhibits lipid raft structural remodeling driven by dysregulated lipid metabolism, which fosters multiple immune escape mechanisms through exosome-mediated immunosuppressive signaling, promotion of tumor-associated macrophage (TAM) M2 polarization, enhanced infiltration of regulatory T cells (Tregs), and functional exhaustion of effector cells, such as CD8^+^ T cells and NK cells. Finally, we discuss targeted therapeutic strategies based on lipid raft characteristics and CRC molecular profiles, proposing an innovative multidimensional treatment framework combining immune checkpoint inhibitors with lipid raft-targeted interventions and chemoradiotherapy. This approach provides theoretical and strategic support for overcoming CRC immunotherapy resistance and advancing clinical translation.

## 1. Introduction

Colorectal cancer (CRC) ranks as the third most common cancer type and the second leading cause of cancer-related deaths worldwide [1], primarily due to metastasis and drug resistance. Over 80% of metastatic CRC patients succumb to the disease within five years of diagnosis [2]. In recent years, immunotherapy represented by immune checkpoint inhibition (ICI) has demonstrated substantial therapeutic potential in various solid tumors [3,4], significantly improving survival rates and clinical outcomes for CRC patients. In 2017, the U.S. Food and Drug Administration approved ICI therapy for treating microsatellite instability-high (MSI-H) tumors, including CRC [5]. By targeting cytotoxic T-lymphocyte antigen 4 (CTLA-4) on T cells, programmed cell death protein 1 (PD-1), and programmed death-ligand 1 (PD-L1) on tumor cells, this approach disrupts immunosuppressive signaling pathways and restores T-cell-mediated antitumor activity [4]. This process is accompanied by remodeling of the tumor microenvironment (TME) and systemic immune modulation. However, such therapeutic efficacy is largely confined to MSI-H CRC patients. Approximately 85% of CRC cases are microsatellite stable (MSS), with a response rate to single-agent ICI below 5% [6]. MSS-type CRC typically exhibits low neoantigen burden and “cold tumor” characteristics, including sparse infiltrating effector T cells with functional exhaustion [5], increased infiltration of immunosuppressive cells, such as regulatory T cells (Tregs) [2], and elevated PD-L1 expression [7], collectively fostering a profoundly immunosuppressive state. Consequently, overcoming the “immune-excluded” or “immune-cold” phenotype of MSS-type CRC and enhancing its sensitivity to immunotherapy remain critical challenges in both clinical practice and basic research.

Emerging studies suggest that lipid rafts, critical membrane microdomains on tumor cell surfaces enriched with immune-related receptors such as PD-L1, CTLA-4, lymphocyte-activation gene 3 (LAG-3), T-cell immunoglobulin, and mucin domain-containing protein 3 (TIM-3), and signaling molecules, such as major histocompatibility complex class I (MHC-I), CD86, and CD47, play pivotal roles in immune cell activation, antigen recognition, immune synapse formation, and functional regulation of immune checkpoint molecules [8,9,10,11]. The lipid raft-mediated signaling network may serve as a novel therapeutic avenue for reshaping the immunosuppressive microenvironment of “cold tumors.”

## 2. Biological Basis of Lipid Rafts

### 2.1. Properties and Composition of Lipid Rafts

At the 2006 Keystone Symposium, lipid rafts were formally defined as small, cholesterol and sphingolipid-enriched membrane domains characterized by high dynamism and heterogeneity [12]. Typically, lipid rafts range in diameter from 10 to 200 nm. Their composition includes major sphingolipids, such as sphingomyelin and gangliosides, with cholesterol intercalating between sphingolipids and, in cooperation with saturated lipids, establishing a distinct liquid-ordered (Lo) membrane state. This preferential interaction is driven by the saturated hydrocarbon chains of sphingomyelin. Compared with the surrounding liquid-disordered (Ld) phase composed of unsaturated lipids, Lo domains retain substantial lateral fluidity, allowing lipids and proteins to diffuse within the membrane [9]. Despite marked differences in lipid packing, bilayer thickness, rigidity, and permeability, the coexistence of Lo and Ld phases provides a fundamental physical basis for the dynamic assembly of lipid rafts and their biological functions in living cells [8,13]. Cholesterol content is considered a critical determinant of lipid raft formation, as cells regulate raft functionality through cholesterol synthesis, uptake, and metabolism. In summary, lipid rafts are recognized as highly ordered, lipid-enriched functional platforms within the cell membrane.

### 2.2. Types and Structural Proteins of Lipid Rafts

Based on structural characteristics, lipid rafts are classified into flat-type and invaginated-type. Flat-type lipid rafts exhibit a planar architecture enriched with flotillin proteins, while invaginated-type lipid rafts are characterized by caveolin proteins, such as caveolin-1 (CAV-1), caveolin-2, and caveolin-3, which induce plasma membrane invagination to form flask-shaped caveolae, participating in vesicle-mediated endocytosis [14]. Caveolin proteins facilitate the spatial redistribution of membrane proteins and are recognized as one of the primary sites for cholesterol efflux from the plasma membrane. These structural proteins collectively maintain lipid raft stability. Notably, CAV-1 modulates signal transduction of multiple tumor-associated pathways, including those mediated by Src family tyrosine kinases and epidermal growth factor receptor (EGFR), which are closely linked to cancer cell proliferation and migration. Studies reveal that CAV-1 exerts robust anti-proliferative activity in CRC cells, and its expression is significantly downregulated in colorectal cancer patients, suggesting that CAV-1 deficiency may promote tumor progression [15,16]. Clinically, low CAV-1 expression has been proposed as a biomarker for poor prognosis. Furthermore, a reduction in membrane cholesterol content, such as through statin-induced cholesterol depletion, disrupts caveolar structure, underscoring the critical role of cholesterol in maintaining lipid raft integrity.

### 2.3. Physiological Functions of Lipid Rafts

The most prominent function of lipid rafts lies in their ability to compartmentalize and enrich specific membrane components within localized regions, creating highly ordered catalytic platforms that regulate interactions with other membrane constituents, thereby modulating their activity [8,9]. As hubs for signal transduction proteins on the plasma membrane, lipid rafts selectively enrich or exclude diverse proteins and kinases, including scaffold proteins, such as glycosylphosphatidylinositol (GPI)-anchored proteins, caveolins, and flotillins; signaling proteins, such as Src family kinases; immune receptor proteins, such as PD-L1 and T-cell receptors (TCRs); cholesterol-binding proteins; and palmitoylated proteins [17]. These components coalesce through lipid–lipid, protein–lipid, and protein–protein interactions to establish compartmentalized signaling pathways [18], which enhance or suppress signal transduction of cell surface receptors in a highly efficient and specific manner. This mechanism promotes activation of signaling cascades while preventing crosstalk between distinct pathways [19].

### 2.4. Lipid Raft-Mediated Immune Signal Transduction

Lipid rafts serve as specialized microdomains for cellular immune responses. Numerous immune regulatory molecules, including TCR, co-stimulatory molecules, such as CD28, immune checkpoint molecules, such as PD-L1 and CD47, and key signaling proteins, such as Lck, Fyn, LAT, and ZAP-70 [20], are enriched within lipid rafts, collectively forming an efficient immune activation platform. Lipid rafts enhance the efficiency and stability of signal transduction by facilitating rapid spatial clustering and activation of signaling molecules. For instance, during T-cell activation, TCRs relocate to lipid raft regions upon recognition of peptide–MHC complexes, forming an “immune synapse.” Within rafts, Lck mediates tyrosine phosphorylation of CD3, subsequently recruiting ZAP-70 and activating scaffold proteins, such as LAT and PLCγ1. This cascade further activates downstream signaling pathways, such as PI3K/Akt and MAPK [21,22], ultimately driving cytokine secretion and T-cell proliferation.

In lipid raft-regulated immune responses, the spatial localization of the immune checkpoint molecule PD-L1 is profoundly influenced by raft architecture. PD-L1, a critical immune checkpoint, suppresses T-cell activation and proliferation by binding to its receptor PD-1 on T cells, thereby promoting tumor immune escape. Studies demonstrate that palmitoylation modification at the Cys272 residue of the PD-L1 cytoplasmic tail facilitates its enrichment in lipid rafts, as palmitoylation significantly enhances membrane protein affinity for rafts [23]. Furthermore, PD-L1 exhibits strong co-localization with two canonical lipid raft markers, GPI-anchored proteins, and CAV-1. Correspondingly, cholesterol depletion disrupts raft integrity, leading to loss of PD-L1 membrane localization, which implies that PD-L1 may participate in raft assembly and play a pivotal role in lipid raft-mediated immune signaling. Notably, inhibiting PD-L1 palmitoylation also blocks its raft localization, offering a potential therapeutic strategy to enhance antitumor immunity [24].

### 2.5. Lipid Raft-Mediated Cell Death

**Figure 1:**
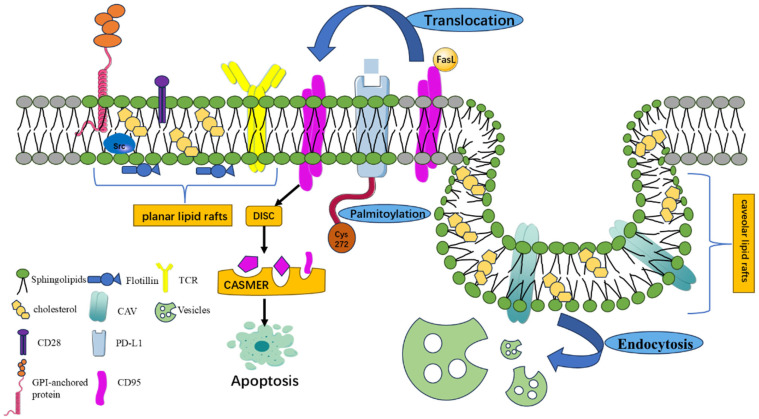
Structural and functional features of lipid rafts. Figure legend: Lipid rafts are highly ordered membrane microdomains enriched in cholesterol and sphingolipids and can be categorized into planar and caveolar rafts based on their structural characteristics. The illustration depicts lipid rafts as functional catalytic platforms that selectively enrich diverse membrane proteins, including GPI-anchored proteins, CAV, flotillin, Src family kinases, and immune receptors, such as PD-L1 and the TCR. Notably, palmitoylation of PD-L1 at Cys272 facilitates its localization within raft domains. Under external stimulation, lipid rafts undergo dynamic reorganization—for example, Fas translocates into raft regions upon ligand engagement, promoting the assembly of the DISC and subsequent formation of the CASMER platform, ultimately driving apoptotic signaling. The schematic also highlights the role of caveolar rafts in receptor endocytosis and vesicular trafficking. Overall, the figure emphasizes that lipid rafts regulate membrane protein enrichment and spatial organization to compartmentalize signaling pathways, thereby orchestrating processes such as vesicle transport, immune responses, and cell death regulation.

Lipid rafts participate in regulating cell death signaling pathways. Apoptosis-related death receptors and their downstream signaling complexes, such as the death-inducing signaling complex (DISC), are often localized within lipid rafts or recruited to rafts upon stimulation, facilitating the initiation and amplification of apoptotic signals. Among these, the Fas receptor (CD95) is the most extensively characterized. Recent studies indicate that CD95 functionality is not only regulated by its expression level but also critically influenced by its spatial localization within membrane microdomains. In 2001, it was first demonstrated that edelfosine induces CD95 translocation into lipid rafts of leukemia cells, leading to co-aggregation of CD95 with raft components and subsequent induction of cancer cell apoptosis [25]. Upon ligand stimulation, CD95 relocates from non-raft regions to lipid rafts. When the local concentration of pro-apoptotic molecules within raft microdomains reaches a threshold, a cluster of the apoptotic signaling molecule-enriched raft (CASMER) is formed, which activates downstream caspase cascades and triggers programmed cell death [11,26]. This spatial reorganization is essential for CD95-mediated apoptotic signaling. Clinical trials confirmed that several antitumor agents with high cholesterol affinity, such as resveratrol and perifosine, can recruit death receptors (CD95, TRAIL, DR4, and DR8) into lipid rafts by disrupting cholesterol–sphingolipid interactions [27,28]. These findings suggest that modulating apoptotic signaling through lipid raft manipulation may represent a promising anticancer strategy (Figure 1).

## 3. Immune Microenvironment Heterogeneity Between MSS and MSI-H Subtypes and Relevance to Lipid Rafts

Tumor immune microenvironment (TIME) of CRC exhibits significant molecular subtype heterogeneity, which directly influences responses to immunotherapy. Based on microsatellite stability status, CRC is classified into the immune-inflammatory MSI-H subtype (approximately 15% of cases) and the immune-excluded MSS subtype (approximately 85% of cases). Key distinctions in their TIMEs are outlined below.

MSI-H CRC is typically characterized by a high tumor mutational burden, generating abundant neoantigens. These neoantigens are presented via MHC-I molecules, activating effector T cells [29]. Consequently, MSI-H tumors exhibit enhanced immunogenicity. Additionally, these tumors are densely infiltrated with immune cells. Elevated chemokine levels such as CXCL9, CXCL10, and CXCL11 recruit and activate tumor-infiltrating lymphocytes (TILs, including CD8^+^ T cells and helper T cells), dendritic cells (DCs), natural killer (NK) cells, and other effector populations. These cells secrete cytokines, such as IFN-γ and TNF-α, along with cytotoxic molecules, such as perforin, directly eliminating tumor cells and amplifying immune responses [30]. Studies demonstrate that high neoantigen load, robust lymphocyte infiltration, and elevated TIL density in MSI-H tumors correlate with prolonged patient survival [31]. These features explain the heightened sensitivity of MSI-H CRC to immune checkpoint therapy.

**Figure 2:**
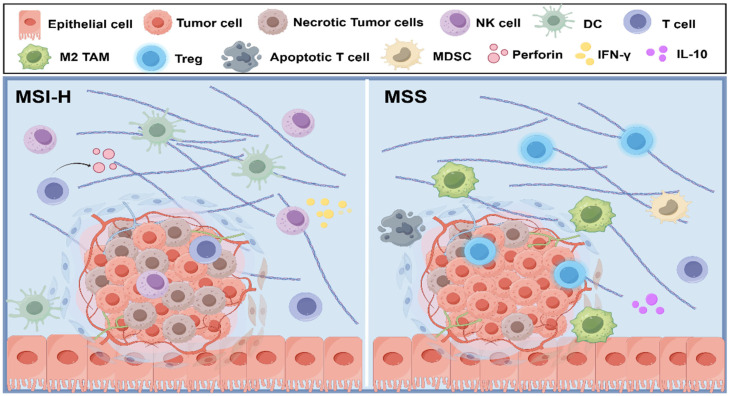
TME differences between MSI-H and MSS-type CRC. Figure legend: MSI-H tumors exhibit abundant tumor-infiltrating lymphocytes, including activated T cells, NK cells, and DCs, accompanied by robust cytotoxic activity, as well as enhanced tumor cell apoptosis and necrosis. In contrast, MSS tumors display reduced effector immune cell infiltration and are enriched with immunosuppressive populations, such as M2 TAMs, Tregs, and MDSCs, resulting in impaired antitumor immunity.

In contrast, MSS-type CRC presents an opposing landscape. Its TIME is dominated by immunosuppressive myeloid cells, including abundant myeloid-derived suppressor cells (MDSCs) and M2-polarized macrophages, accompanied by exhausted CD8^+^ T cells and excessive Tregs infiltration [32]. Concurrently, aberrant activation of specific signaling pathways exacerbates immunosuppression. For instance, hyperactivation of the Wnt/β-catenin pathway downregulates chemokines CCL4 and CCL5, impairing DC precursor migration to tumor sites and thereby reducing antigen presentation and T-cell activation. Similarly, elevated transforming growth factor-β (TGF-β) signaling promotes fibroblast differentiation into cancer-associated fibroblasts (CAFs) and induces Treg expansion. These cells release immunosuppressive factors, such as IL-10, and mediate tissue fibrosis, further limiting effector T-cell infiltration into tumors [33]. Collectively, MSS-type CRC establishes a tightly immunosuppressive microenvironment, leading to poor ICI efficacy (Figure 2).

To overcome the immune-cold phenotype exhibited by MSS-type CRC, an emerging strategy involves modulating the structure and functionality of lipid rafts on tumor cell membranes. Numerous cancer-associated molecules co-localize with lipid rafts, such as growth factor receptors that drive tumor proliferation, the cell adhesion receptor CD44 that facilitates migration, vascular endothelial growth factor receptors mediating angiogenesis and metastasis [8], and Src family protein tyrosine kinases responsible for early CRC tumor cell adhesion [34,35]. If pharmacological interventions can disrupt lipid raft-dependent signaling cascades, this approach holds promise for reversing the immunosuppressive microenvironment in CRC and enhancing the sensitivity of MSS-type patients to PD-1/PD-L1 antibody therapy.

It is widely accepted that alterations in membrane lipid composition serve as key drivers of functional changes in lipid rafts. MSS-type CRC is frequently associated with dysregulated lipid metabolism, where cancer cells exhibit marked affinity for cholesterol, accompanied by significantly elevated levels of cholesterol and sphingolipids [36]. This leads to aberrant structural remodeling of lipid rafts, resulting in either loss of function or pathological activation. Substantial evidence demonstrates that lipid rafts are extensively involved in CRC cell survival and proliferation-related signaling pathways. For instance, epigallocatechin gallate, a bioactive compound in green tea, inhibits tumor growth by disrupting CRC cell membrane rafts, thereby suppressing EGFR signaling [37]. Similarly, activation of the insulin-like growth factor system and the PI3K/AKT pathway depends on raft integrity. CRC cells maintain proliferative, migratory, invasive, and immune-evasive capacities by activating the PI3K/AKT/mTOR pathway to upregulate intracellular cholesterol levels. Cholesterol-depleting agents disrupt rafts, causing translocation of insulin-like growth factor-1 receptors from raft to non-raft regions, which prevents effective recruitment and phosphorylation of AKT within rafts, thus inhibiting downstream pro-growth signaling [38]. Additionally, lipid rafts mediate enhanced secretion of angiogenic factors, such as vascular endothelial growth factor (VEGF), by tumor cells. The localization of Hsp90 to lipid rafts has been identified as essential for STAT3 activation and VEGF-driven signaling in CRC cells [39]. These findings underscore the functional centrality of lipid rafts in oncogenic signaling.

Immune dysfunction in CRC is closely linked to tumor cell overexpression of PD-L1. The stability of PD-L1 in cancer cells is primarily regulated by post-translational modifications, such as deubiquitination, glycosylation, or lysosomal degradation, as well as palmitoylation [40,41]. Zhang et al. demonstrated that benzobactin C enhances T-cell antitumor immunity by targeting DHHC3 to inhibit PD-L1 palmitoylation [42]. Furthermore, abundant cholesterol in cancer cells directly binds to the transmembrane domain of PD-L1 via two CRAC motifs, forming a sandwich-like structure that stabilizes PD-L1 on membrane rafts and protects it from proteasomal degradation [43]. This evidence indicates that the cholesterol-enriched milieu of CRC lipid rafts amplifies PD-L1-mediated immunosuppression. Beyond PD-L1, other immunoregulatory proteins are enriched in CRC rafts. For example, overexpression of the membrane protein STOML2 in rafts activates the NF-κB pathway, driving the upregulation of immune- and proliferation-related genes such as PD-L1, CCND1, and VEGF [44].

As discussed above, a cholesterol-enriched microenvironment is generally regarded as protumorigenic. Paradoxically, however, lipid raft enrichment driven by elevated cholesterol levels also creates opportunities to induce apoptosis. Adequate cholesterol not only provides optimal membrane fluidity to facilitate clustering of apoptosis-associated receptors but also functionally isolates these apoptotic signal clusters from interference by pro-survival signaling. Consequently, lipid rafts play dual roles in CRC tumor biology: they potentiate proliferation- and survival-related signaling while simultaneously aggregating apoptotic receptors or immunosuppressive molecules. These functional dichotomies position lipid rafts as a potential therapeutic target for anticancer interventions. In the following sections, we will elaborate on the regulatory mechanisms of lipid rafts in immune cell functionality and explore potential raft-targeted therapeutic strategies.

## 4. Systemic Regulation of Immune Cell Function by Lipid Rafts

### 4.1. Exosome-Mediated Immunosuppression

Exosomes are membrane vesicles with a diameter of 50–150 nm, generated and released by cells via the exocytic pathway. Exosome biogenesis is closely linked to lipid rafts—they originate from raft-associated invaginated vesicles (early endosomes) and exhibit membranes enriched in lipid raft-like components, such as cholesterol and GM1 ganglioside [45,46]. Their luminal cargo includes membrane-associated molecules, such as surface receptors, MHC molecules, adhesion molecules, tetraspanins, and immune mediators, such as protein kinases, chemokines, and heat shock proteins [47]. Exosomes transmit intercellular signals by activating downstream pathways through ligand–receptor interactions on target cells.

Tumor cells exhibit significantly higher exosome production than normal cells. Exosomes secreted by CRC cells (termed tumor-derived exosomes, Tex) contain heat shock protein 70, MHC-I molecules, and native tumor antigens, conferring immunogenic potential. Tex transfers tumor antigens to antigen-presenting cells (APCs), thereby priming T-cell-mediated antitumor immunity [48]. Early studies reported that combining exosomes isolated from ascites of advanced CRC patients (Aex) with granulocyte–macrophage colony-stimulating factor enhances systemic antitumor immunity and cytotoxic T-lymphocyte (CTL) responses [49], suggesting context-dependent immunostimulatory roles of Tex. However, accumulating evidence highlights the dominant immunosuppressive effects of tumor-derived exosomes. Tex is enriched with immunosuppressive cytokines (e.g., prostaglandin E2 [PGE2], IL-10, TGF-β), membrane-associated death ligands (e.g., FasL, TRAIL), and other factors that induce apoptosis of activated CD8^+^ T cells, impair functional DC differentiation, suppress NK cell cytotoxicity by downregulating perforin expression, and promote proliferation of MDSCs and Tregs [50,51,52]. Tumor exosomes internalized by host cells upregulate phosphorylation of STAT3 and SMAD2/3 in recipient tissues, accompanied by partial excessive secretion of IL-10 and TGF-β1, thereby reshaping local and distant microenvironments to facilitate immune evasion [53].

Mechanistic analysis reveals that exosome production critically depends on lipid raft formation in donor cell membranes. Exosomal membranes are enriched in cholesterol and ceramide, the latter being a sphingolipid metabolite generated by neutral sphingomyelinase (nSMase) mediated hydrolysis of sphingomyelin, which is considered to play a critical role in the process of exosome budding [54]. Exosomes also harbor tetraspanins, including CD37, CD63, and CD81, which are canonical lipid raft markers [55]. Furthermore, many raft-associated proteins directly participate in endocytic budding and cargo sorting, while others are packaged into exosomes to serve as signaling molecules. For instance, flotillins, components of endocytic “floating microdomains,” facilitate cargo loading during exosome biogenesis and cooperate with active RAB31 to trigger vesicle budding [56]. Conversely, CAV-1, as one of the lipid raft markers, suppresses exosome formation by inhibiting nSMase activity and reducing membrane ceramide levels [57]. These findings suggest that targeting lipid raft-associated pathways may modulate exosome secretion. Pharmacologically targeting lipid rafts could reduce tumor exosome release, thereby diminishing immunosuppressive molecule accumulation in the TIME and suppressing the recruitment and activation of inhibitory cells. This strategy holds promise for partially reversing the protumorigenic effects mediated by Tex. Consequently, the lipid raft–exosome axis represents a burgeoning focus in tumor immunomodulation research and a unique therapeutic avenue to counteract immune evasion.

### 4.2. Lipid Raft-Mediated Dysregulation of Immune Cell Function

Lipid rafts critically regulate the differentiation and functionality of diverse immune cells, including innate and adaptive immune populations. CRC tumors usually disrupt lipid raft structures on immune cell membranes to suppress antitumor immunity while fostering immunosuppressive components, thereby establishing an immune-evasive microenvironment. Subsequently, we delineate the mechanisms by which lipid rafts modulate functions in TAMs, Tregs, and other effector immune cells.

#### 4.2.1. Polarization of Tumor-Associated Macrophages

TAMs constitute a critical immune cell population within the TIME, playing a central role in tumor-associated inflammation and immune regulation. TAMs are functionally polarized into two major subtypes: antitumor M1-like and protumorigenic M2-like phenotypes. Current understanding posits that tumor-secreted factors such as TGF-β, IL-10, and macrophage colony-stimulating factors drive the differentiation of monocytes–macrophages toward the M2 phenotype. M2-polarized TAMs abundantly express immunosuppressive molecules (e.g., VEGF, PD-L1, IL-10) and metabolites (e.g., indoleamine 2,3-dioxygenase, PGE2), which suppress effector T-cell proliferation [58], induce NK cell dysfunction, and secrete matrix metalloproteinases to activate CAFs. This cascade promotes extracellular matrix remodeling and CXCL12-mediated recruitment of immunosuppressive cells, establishing a self-reinforcing immunosuppressive network. Additionally, VEGF overexpression by M2 TAMs stimulates tumor angiogenesis [59], enhancing cancer cell invasiveness and metastatic potential. Although molecular drivers of TAM polarization vary across malignancies, altered membrane cholesterol levels, which are a key determinant of lipid raft integrity, are widely recognized to modulate TAM polarization states, suggesting a mechanistic link to lipid raft structural dynamics.

**Figure 3:**
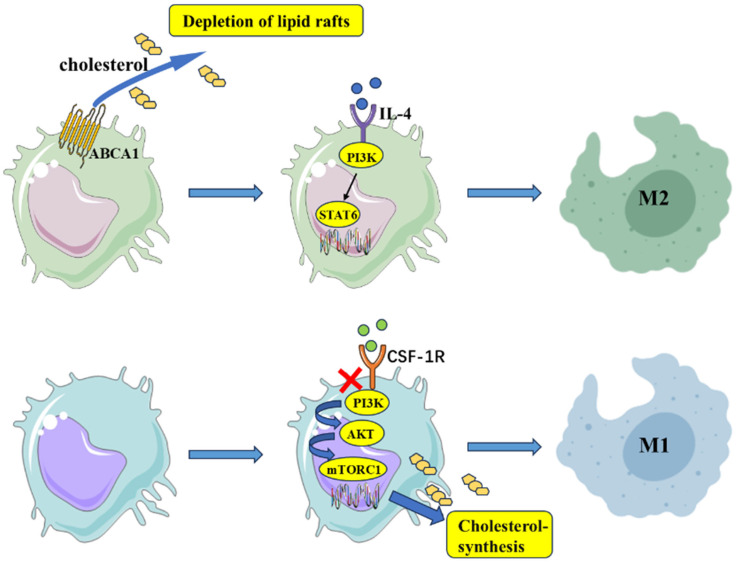
Cholesterol metabolism regulates TAM polarization. Figure legend: This figure illustrates that cholesterol availability and lipid raft integrity critically influence macrophage polarization within the tumor microenvironment. Cholesterol efflux mediated by ABCA1 leads to lipid raft depletion, thereby impairing IL-4–induced activation of the PI3K–STAT6 pathway and driving macrophages toward an M2-like phenotype. Blockade of the CSF-1R receptor results in inhibition of the PI3K–AKT–mTORC1 signaling cascade and a reduction in macrophage intrinsic cholesterol synthesis, thereby promoting M1 polarization.

Emerging evidence indicates that manipulating cholesterol metabolism in TAMs can redirect their polarization. On one hand, inhibiting cholesterol biosynthesis suppresses M2 polarization. For example, the novel colony-stimulating factor 1 receptor pathway inhibitor PXB17 blocks the PI3K-AKT-mTORC1 signaling axis, downregulates cholesterol synthase expression, depletes intracellular cholesterol, and reprograms M2 TAMs toward an M1-like phenotype. This shift enhances CD8^+^ T-cell-mediated antitumor immunity and suppresses CRC cell progression [60]. Conversely, excessive cholesterol efflux may exacerbate M2 polarization. Toby et al. demonstrated that ATP-binding cassette (ABC) transporter-mediated cholesterol depletion from TAM membranes disrupts raft architecture, paradoxically amplifying IL-4-driven TAM reprogramming and protumorigenic functions [61]. Thus, maintaining balanced TAM membrane cholesterol levels appears essential for preserving antitumor activity. Targeting cholesterol efflux mechanisms, such as inhibiting ABC transporter activity or neutralizing high-density lipoprotein, has been proposed as a potential strategy to stabilize TAM lipid rafts and counteract tumor-induced M2 polarization. In preclinical models of MSS-type CRC, such approaches demonstrate dual therapeutic benefits: alleviating the immune-cold TIME and synergizing with anti-PD-1 therapy to enhance treatment efficacy [60] (Figure 3).

#### 4.2.2. Infiltration and Functional Enhancement of Regulatory T Cells

In addition to TAMs, CD4^+^CD25^+^FoxP3^+^ Tregs are also markedly enriched in the TIME of CRC. Tregs suppress antitumor immunity through multiple mechanisms, and their infiltration levels correlate with poor prognosis in most solid tumors. However, the role of Tregs in CRC remains debated. Some studies have paradoxically observed that high FoxP3^+^ Treg density in tumor tissues associates with improved patient survival, potentially due to Treg-secreted IL-10 suppressing gut microbiota-driven chronic inflammation and reducing polyp formation, thereby indirectly inhibiting tumor progression [62,63]. Nonetheless, the consensus underscores Tregs’ predominant role in promoting tumor immune evasion. In CRC patients, Treg overexpress immunosuppressive molecules such as TIM-3, LAG-3, TGF-β, and IL-10. Elkord et al. demonstrated that CRC tumors induce conventional CD4^+^ T cells to upregulate multiple immune checkpoint receptors, thereby amplifying tumor immune escape [64].

CRC cells drive Treg enrichment via lipid raft-associated signaling pathways. Under the regulation of lipid raft-mediated signals, such as PI3K/Akt and TGF-β, CRC cells secrete elevated levels of immunosuppressive cytokines, such as TGF-β and IL-10, which polarize peripheral conventional CD4^+^ T cells toward a Treg phenotype. Furthermore, tumor cells express chemokine CCL4 to recruit circulating Tregs into tumor sites [65]. Once within the TIME, Tregs suppress immune responses through diverse mechanisms, including inhibition of IFN-γ production by CD8^+^ T cells, disruption of IL-2 metabolism, adenosine release, induction of tolerogenic DC phenotypes, and CTLA-4-mediated attenuation of APC co-stimulatory capacity [63]. Notably, lipid raft integrity in Tregs is directly linked to their immunosuppressive functionality. Recent studies reveal that modulators of cellular cholesterol metabolism influence Treg survival and activity. For instance, proprotein convertase subtilisin/kexin type 9, a protease inhibiting cholesterol clearance, promotes Treg apoptosis upon blockade by depriving cells of cholesterol, thereby alleviating Treg-mediated immunosuppression and enhancing the efficacy of anti-PD-1 therapy [66]. These findings suggest that lipid rafts serve as critical scaffolds for maintaining Treg function, implying that targeting raft-related metabolic pathways could attenuate Treg-driven immune suppression.

#### 4.2.3. Exhaustion of Effector Cells

Within the complex immune microenvironment of CRC, lipid rafts exert profound yet dualistic effects on antitumor effector cells, such as CTLs and natural NK cells. While lipid raft integrity is essential for effector cell activation under physiological conditions, tumors exploit raft disruption to impair effector cell functionality, driving exhaustion or inactivation.

CD8^+^ T-Cell Dysfunction. TCR signaling critically depends on lipid raft integrity, as previously discussed. Disruption of T-cell membrane rafts (for instance, through cholesterol depletion) severely compromises TCR clustering and signal transduction. Even under antigenic stimulation, CD8^+^ T cells fail to fully activate and progressively adopt an exhausted phenotype [67]. Some factors of the tumor microenvironment, including persistent antigen exposure and metabolite accumulation, may perturb T-cell membrane lipid composition. Emerging evidence suggests that elevated cholesterol levels exacerbate endoplasmic reticulum (ER) stress, wherein the ER stress sensor X-box binding protein 1 upregulates inhibitory receptors (PD-1, LAG3) on CD8^+^ T cells [2], accelerating functional exhaustion.

NK Cell Impairment. Lipid rafts also govern NK cell-mediated tumor cytotoxicity. Effective NK cell recognition requires stable expression of NKG2D ligands (MHC class I chain-related proteins A/B) on tumor cell surfaces [68]. These ligands must cluster within tumor membrane rafts to evade endocytic degradation and persistently display “kill me” signals [69]. CRC cells subvert this process by reprogramming cholesterol metabolism or altering membrane lipid architecture, thereby disrupting lipid raft microdomains. This disruption facilitates the shedding or clearance of NKG2D ligands, ultimately reducing NK cell-mediated tumor recognition [70,71]. Meanwhile, the formation of functional immunological synapses by NK cells is also dependent on cholesterol-enriched lipid rafts. Insufficient membrane cholesterol disrupts receptor clustering, thereby impairing NK cell-mediated cytotoxicity against target cells.

Metabolic Competition in the TIME. A recent study uncovered a novel immune evasion strategy wherein tumors hijack metabolic resources to disrupt effector cell rafts. Specifically, CD16^+^ neutrophil subsets enriched in the CRC TIME competitively scavenge extracellular cholesterol, depriving NK cells of cholesterol necessary for raft assembly. Cholesterol-starved NK cells exhibit defective intracellular cytotoxic signaling and markedly reduced antitumor activity [72]. This mechanism highlights how tumors exploit “metabolic competition” to sabotage raft-dependent effector cell functions and evade immune destruction.

It is imperative to note that lipid raft integrity further influences tumor apoptosis pathways initiated by effector cells. As previously discussed, death receptors, such as Fas and TRAIL, critically mediate immune cell–tumor cell interactions. The membrane clustering efficiency and signaling transduction of these receptors in tumor cells are tightly regulated by raft stability. Widespread raft disruption may prevent the formation of Fas-mediated extracellular apoptotic signaling cascades, partially compromising the efficacy of effector cell-induced tumor cell apoptosis.

Thus, lipid rafts in CRC immunoregulation function as dual-edged regulators, serving as “amplifiers” for immunosuppressive cells and “impediments” for effector immune cells. By strategically manipulating raft microdomains, tumors amplify inhibitory signaling in TAMs and Tregs while attenuating activation signals in CD8^+^ T cells and NK cells. This bidirectional dysregulation disrupts immune surveillance and tumor clearance mechanisms. Accumulating evidence positions lipid raft dysfunction as a central orchestrator of immune dysregulation in CRC.

## 5. Immunotherapeutic Strategies Targeting Lipid Rafts

As previously discussed, the critical role of lipid rafts in regulating immune cell functionality and remodeling the TIME of CRC has positioned them as a promising target in cancer immunotherapy. Targeted interventions aimed at modulating lipid raft structure or their upstream regulatory pathways have increasingly demonstrated potent immunostimulatory and antitumor potential. The following section summarizes three representative strategies currently under investigation.

### 5.1. Strategies Targeting Cholesterol Metabolism

Given the critical dependence of lipid raft stability on cholesterol levels, reducing membrane cholesterol content is a well-established strategy to disrupt raft architecture and interfere with raft-mediated signaling. Statins represent the most classical therapeutic agents in this category. Retrospective studies indicate that statins prolong survival in CRC patients receiving first-line chemotherapy [73]. These drugs inhibit 3-hydroxy-3-methylglutaryl-CoA (HMG-CoA) reductase activity to block cholesterol biosynthesis, thereby depleting membrane cholesterol, destabilizing lipid rafts, and impairing the stability of membrane-associated signaling complexes. In CRC models, statins suppress pro-survival pathways, such as AKT, alleviate T-cell immunosuppression, and induce tumor cell apoptosis [74]. Intriguingly, cholesterol synthesis inhibition reduces ER stress, downregulating PD-1 expression on CD8^+^ T cells and mitigating T-cell exhaustion [75]. Correspondingly, cholesterol reduction may indirectly downregulate PD-L1 expression in tumor cells. Collectively, these effects synergize to enhance antitumor immunity. Beyond statins, cholesterol-sequestering agents, such as methyl-β-cyclodextrin (MβCD), also disrupt lipid rafts and augment immune activation [76].

The distribution and homeostasis of membrane cholesterol are regulated by multiple transporters, among which ABC transporters, such as ATP-Binding Cassette Subfamily-A Member 1 (ABCA1), play central roles in cholesterol efflux and raft dynamics. ABCA1 overexpression correlates with increased recurrence risk in stage I–III CRC patients [77]. It has been found by Aguirre et al. that ABCA1 upregulation drives epithelial–mesenchymal transition (EMT), while CAV-1 regulates focal adhesion formation, thereby promoting tumor proliferation and migration [78]. In vitro cell experiments confirm that MβCD treatment significantly undermines the migratory capacity of ABCA1-overexpressing CRC cells [78]. Consequently, ABC transporter expression profiles may influence the sensitivity to raft-targeted therapies. Cells with pronounced cholesterol accumulation exhibit heightened raft-dependent signaling, rendering them more susceptible to lipid raft disruption. This rationale underpins the proposal to utilize ABC transporter expression patterns as biomarkers for personalized raft-targeted interventions.

Notably, the colorectum serves as a crucial interface for host and microbiota metabolic crosstalk, prompting exploration of gut microbiota in raft modulation strategies. For instance, certain microbial metabolites, such as short-chain fatty acids (SCFAs), like butyrate, modulate cholesterol metabolism via dual mechanisms: firstly, suppressing HMG-CoA reductase expression to inhibit host cholesterol synthesis; and secondly, altering sterol regulatory element-binding protein 2 activity to upregulate low-density lipoprotein (LDL) receptor expression, accelerating LDL uptake from circulation. These actions reduce cholesterol levels in intestinal epithelial cell membranes and serum, destabilizing oncogenic signaling and lipid rafts, which may contribute to CRC prevention [79]. Recent studies further reveal that butyrate enhances CD8^+^ T-cell cytotoxicity through metabolic reprogramming [80] and synergizes with radiotherapy in CRC patients without damaging healthy mucosa [81]. Building on these antitumor properties, microbiota remodeling, such as enriching SCFA-producing bacteria, could serve as an adjuvant strategy to potentiate raft-targeted immunotherapies, improving efficacy while minimizing systemic toxicity.

### 5.2. Strategies for Targeted Delivery via Lipid Nanocarriers

Given that lipid rafts are ubiquitously present in normal cells, systemic pharmacological interventions, such as cholesterol-depleting agents, may induce non-specific toxicity due to limited selectivity. Lipid nanoparticles (LNPs), characterized by their uniform lipid cores, have been widely used for the delivery of small molecules and nucleic acids [82]. Therefore, developing drug delivery systems capable of selectively targeting raft microdomains in colorectal cancer cells has emerged as a highly promising therapeutic strategy. The conceptual basis of this nanodelivery platform lies in achieving “dual targeting”, modifying the nanocarrier with raft-affinitive ligands to promote its enrichment within tumor cell raft microdomains and incorporating CRC-specific ligands to enhance cancer cell recognition and thus minimize off-target effects on normal tissues.

To this end, lipid-based nanoparticles, solid lipid nanoparticles, or polymer–lipid hybrid nanocarriers may be co-functionalized with raft-targeting moieties (e.g., cholesterol-binding peptides, anti-Cav-1, or anti-flotillin antibody fragments) and CRC-associated targeting ligands (such as carcinoembryonic antigen, chondroitin sulfate, tumor-homing peptides, RGD peptides, or the EGFR-binding peptide GE11), whose receptors exhibit low or negligible expression in normal cells but are highly expressed in CRC [83]. Studies have shown that such surface modification strategies can significantly enhance the targeting efficiency of nanocarriers. For example, Liu et al. demonstrated that cRGD-modified nanocarriers loaded with anticancer drugs displayed markedly superior inhibitory activity against colon cancer cells compared with free drugs [84].

In addition, the TME of CRC is typically acidic and hypoxic. Thus, designing pH-responsive LNPs that remain stable under physiological conditions but efficiently release their payload in the acidic tumor milieu is of particular importance. Juang et al. developed a peptide-modified pH-sensitive LNP that achieved high drug release efficiency under acidic conditions and exhibited excellent tumor-targeting capability and antitumor efficacy in animal models [85]. Furthermore, rational modulation of nanoparticle size (10–100 nm) and surface charge can enhance penetration through the dense tumor stroma and further promote interactions with lipid rafts on the cell membrane. Coating the nanoparticles with materials, such as chitosan, may additionally improve mucoadhesive properties and prolong retention at tumor sites [86], thereby facilitating enrichment within raft microdomains.

Lipid nanocarriers also possess the capacity for co-delivery of multiple therapeutic agents. They can encapsulate drugs that regulate cholesterol metabolism (such as MβCD, statins, or inhibitors of cholesterol biosynthesis) to locally disrupt raft integrity in tumor cells, thereby inducing immunogenic cell death, enhancing antigen presentation, and promoting antitumor immune responses [76]. Moreover, co-delivery of conventional chemotherapeutic agents (e.g., 5-fluorouracil or platinum-based drugs), immunomodulators, or gene therapeutics (including TLR/STING agonists, siRNAs, or antisense oligonucleotides) not only facilitates their more efficient entry into tumor cells but also enables them to exert their activity in the context of raft disruption. This combinatorial approach further targets raft-associated immunosuppressive pathways (e.g., PD-L1, CTLA-4, TGF-β, and Wnt), thereby amplifying local immune responses. Previous studies have demonstrated that intratumoral delivery of TLR or STING agonists can reduce PD-L1 expression and strengthen antitumor immunity [87]. Additionally, GE11-modified core–shell LNPs co-delivering FOLFOX and siPD-L1 have shown significant tumor-inhibitory effects in CRC mouse models [88]. Collectively, although research on this dual-targeting strategy—combining lipid raft targeting with CRC tumor-specific targeting—remains relatively limited, lipid-based nanocarriers offer a promising strategy for precise modulation of raft microdomains and associated signaling pathways, with the potential to enhance therapeutic efficacy while minimizing systemic toxicity. Studies focusing on raft-specific delivery and dual-targeting strategies remain relatively limited, and further investigation is required to refine and substantiate their therapeutic potential.

### 5.3. Lipid Raft Heterogeneity and Immunotherapy Response

**Figure 4:**
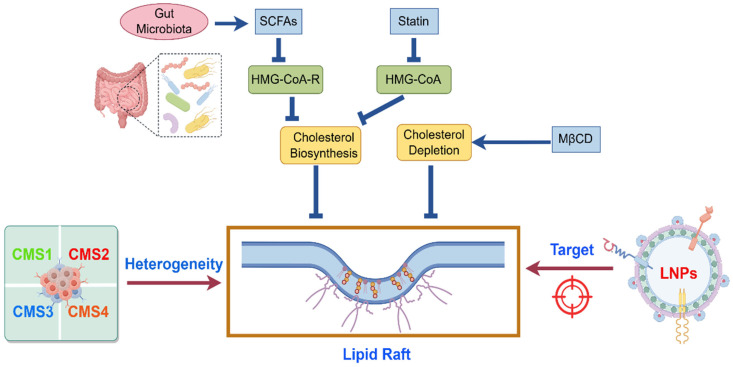
Therapeutic strategies of targeting lipid rafts in CRC. Figure legend: Gut microbiota-derived SCFAs and statins modulate cholesterol biosynthesis via the HMG-CoA pathway, while MβCD depletes membrane cholesterol, all of which impact tumor cell lipid raft structures. LNPs can be designed to selectively target raft-enriched molecules and modulate raft-dependent signaling. Notably, lipid rafts vary in abundance, distribution, and protein composition across CMS subtypes of CRC, highlighting the need for personalized raft-targeting strategies.

Significant heterogeneity exists in the composition and distribution of lipid rafts on tumor cell membranes across patients, molecular subtypes, and even distinct regions within the same tumor. Based on transcriptional heterogeneity, CRC can be classified into four consensus molecular subtypes (CMS): CMS1 (MSI-Immune) is characterized by high microsatellite instability (MSI-H), high tumor mutational burden, and strong immune activation; CMS2 (Canonical) displays an epithelial phenotype with active WNT/MYC signaling and chromosomal instability; CMS3 (Metabolic) is dominated by metabolic reprogramming, including altered glucose, lipid, and amino acid metabolism, often accompanied by KRAS mutations; and CMS4 (Mesenchymal) exhibits pronounced stromal activation, EMT, abundant CAF, and strong TGF-β signaling [89]. Notably, more than half of CRC tumors display transcriptional features of multiple CMS groups and are, therefore, categorized as “mixed CMS” [90]. Studies report that CMS4-type cells are predominantly localized to invasive tumor margins, whereas other CMS subtypes are more frequently distributed in the tumor core [91]. Compared to CMS1-type tumors, CMS4-type tumors exhibit reduced antitumor T-cell infiltration and elevated Treg proportions, suggesting that lipid rafts at invasive margins may preferentially cluster immunosuppressive receptors, while those in the tumor core may favor receptors associated with immune activation. Such spatiotemporal heterogeneity likely impacts immunotherapy efficacy. Tumor cells in certain regions may retain immune escape capabilities via residual lipid rafts even after pharmacological raft disruption, posing substantial challenges for CRC treatment. To address this, future studies should integrate single-cell sequencing, multiplexed imaging, and spatial lipidomics to delineate raft composition and functional states across cell types and tumor regions. This precision profiling will identify raft-targeting-sensitive cellular subpopulations, enabling the design of much more tailored combination therapies to improve overall efficacy (Figure 4).

## 6. Conclusions

In summary, lipid rafts play a critical role in CRC by selectively enriching key signaling molecules that regulate tumor progression, therapeutic resistance, and immune evasion. Modulating raft composition or stability has been shown to effectively remodel the CRC immune microenvironment. Future strategies may overcome immunotherapy resistance through combinational approaches, for example, combining PD-1/PD-L1 blockade with interventions that regulate membrane cholesterol metabolism or inhibit PD-L1 enrichment within rafts, thereby synergistically relieving tumor-mediated immunosuppression and improving the efficacy of CRC immunotherapy.

**Figure 5:**
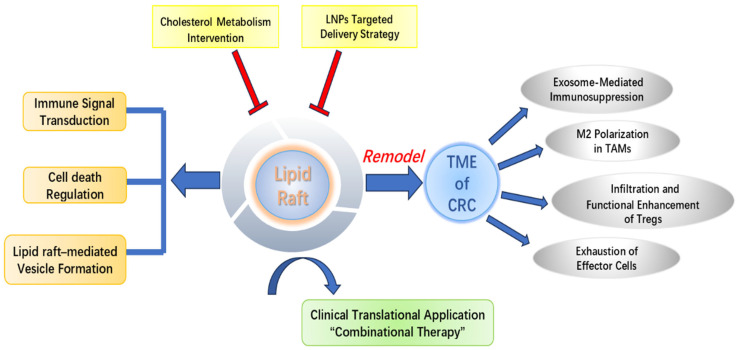
Lipid rafts in a CRC immune microenvironment: mechanisms and interventions. Figure legend: Lipid rafts participate in remodeling the TIME of CRC by regulating immune signaling, cell death, and vesicle formation, including exosome-mediated immunosuppression, TAM M2 polarization, enhanced Treg function, and effector cell exhaustion. Cholesterol metabolism interventions and targeted delivery via LNPs can modulate lipid rafts, providing potential strategies for clinical translation in CRC combination therapy.

Despite the promising potential of targeting lipid rafts to enhance CRC immunotherapy sensitivity, substantial challenges remain in translating mechanistic insights into clinical applications, underscoring the need for bidirectional integration between basic and clinical research. Current raft-targeting strategies primarily focus on membrane proteins commonly enriched across tumors, such as regulators of cell proliferation, angiogenic factors, and components of immune checkpoint pathways. However, membrane protein profiles vary among tumor types and undergo dynamic remodeling under the influence of the TME, limiting the applicability of generalized interventions to CRC. Thus, it is essential to delineate CRC-specific molecular networks through which rafts regulate immune escape, such as PD-L1 raft enrichment and immune cell dysfunction, to facilitate precise target identification and therapeutic development. Clinical translation is further hindered by the predominance of in vitro and animal studies, with limited evidence regarding the dynamic regulation and therapeutic impact of lipid rafts in humans. To bridge this gap, several strategies may be envisioned: establishing patient-derived organoid autologous immune cell co-culture systems to characterize raft remodeling-mediated reprogramming of TILs using transcriptomic analyses and developing novel raft-tracking probes (e.g., CAV-anchored fluorescent reporters) to visualize membrane microdomain dynamics in vivo. Given the rising global incidence and mortality of CRC, future clinical efforts should prioritize optimization of combinational regimens, such as “immune checkpoint inhibition + raft-targeting intervention + conventional chemotherapy/radiotherapy.” Ultimately, bridging the translational divide from bench to bedside will be crucial for realizing the clinical potential of raft-targeted strategies and advancing CRC therapy toward greater precision and personalization (Figure 5).

## Data Availability

No datasets were generated or analysed during the current study.

## Abbreviations

ABC	ATP-binding cassette
ABCA1	ATP-binding cassette subfamily-A member 1
APCs	antigen-presenting cells
CAFs	cancer-associated fibroblasts
CASMER	cluster of apoptotic signaling molecule-enriched raft
CAV-1	caveolin-1
CMS	consensus molecular subtype
CRC	colorectal cancer
CTL	cytotoxic T-lymphocyte
CTLA-4	cytotoxic T-lymphocyte antigen 4
DCs	dendritic cells
DISC	death-inducing signaling complex
EGFR	epidermal growth factor receptor
EMT	epithelial–mesenchymal transition
ER	endoplasmic reticulum
GPI	glycosylphosphatidylinositol
HMG-CoA	3-hydroxy-3-methylglutaryl-CoA
ICI	immune checkpoint inhibition
LAG-3	lymphocyte-activation gene 3
Ld	liquid-disordered
LDL	low-density lipoprotein
LNPs	lipid nanoparticles
Lo	liquid-ordered
MDSCs	myeloid-derived suppressor cells
MHC-I	major histocompatibility complex class I
MSI-H	microsatellite instability-high
MSS	microsatellite stable
MβCD	methyl β-cyclodextrin
NK	natural killer
nSMase	neutral sphingomyelinase
PD-1	programmed cell death protein
PD-L1	programmed death-ligand 1
PGE2	prostaglandin E2
SCFAs	short-chain fatty acids
TAMs	tumor-associated macrophages
TCRs	T-cell receptors
Tex	tumor-derived exosome
TGF-β	transforming growth factor-β
TILs	tumor-infiltrating lymphocytes
TIM-3	T-cell immunoglobulin and mucin domain-containing protein 3
TIME	tumor immune microenvironment
TME	tumor microenvironment
Tregs	regulatory T cells
VEGF	vascular endothelial growth
